# Maintenance of cellular vitamin B_6_ levels and mitochondrial oxidative function depend on pyridoxal 5′-phosphate homeostasis protein

**DOI:** 10.1016/j.jbc.2023.105047

**Published:** 2023-07-13

**Authors:** Jolita Ciapaite, Carlo W.T. van Roermund, Marjolein Bosma, Johan Gerrits, Sander M. Houten, Lodewijk IJlst, Hans R. Waterham, Clara D.M. van Karnebeek, Ronald J.A. Wanders, Fried J.T. Zwartkruis, Judith J. Jans, Nanda M. Verhoeven-Duif

**Affiliations:** 1 Department of Genetics, University Medical Center Utrecht, Utrecht, The Netherlands; 2 United for Metabolic Diseases, The Netherlands; 3 Laboratory Genetic Metabolic Diseases, Amsterdam Gastroenterology & Metabolism, Amsterdam University Medical Centres, University of Amsterdam, Amsterdam, The Netherlands; 4 Department of Genetics and Genomic Sciences, Icahn Institute for Genomics and Multiscale Biology, Icahn School of Medicine at Mount Sinai, New York, New York, USA; 5 Departments of Pediatrics and Human Genetics, Emma Center for Personalized Medicine, Amsterdam University Medical Centres, University of Amsterdam, Amsterdam, The Netherlands; 6 Department of Pediatrics, Centre for Molecular Medicine and Therapeutics, BC Children’s Research Institute, University of British Columbia, Vancouver, British Columbia, Canada; 7 Department of Molecular Cancer Research, Center for Molecular Medicine, Oncode Institute, University Medical Center Utrecht, Utrecht, The Netherlands

**Keywords:** pyridoxal 5′-phosphate homeostasis protein PLPHP (PROSC), pyridox(am)ine 5′-phosphate oxidase (PNPO), vitamin B_6_ metabolism, mitochondrial dysfunction, α-ketoglutarate, transaminases

## Abstract

Recently, biallelic variants in *PLPBP* coding for pyridoxal 5′-phosphate homeostasis protein (PLPHP) were identified as a novel cause of early-onset vitamin B_6_–dependent epilepsy. The molecular function and precise role of PLPHP in vitamin B_6_ metabolism are not well understood. To address these questions, we used PLPHP-deficient patient skin fibroblasts and HEK293 cells and YBL036C (PLPHP ortholog)-deficient yeast. We showed that independent of extracellular B_6_ vitamer type (pyridoxine, pyridoxamine, or pyridoxal), intracellular pyridoxal 5′-phosphate (PLP) was lower in PLPHP-deficient fibroblasts and HEK293 cells than controls. Culturing cells with pyridoxine or pyridoxamine led to the concentration-dependent accumulation of pyridoxine 5′-phosphate and pyridoxamine 5′-phosphate (PMP), respectively, suggesting insufficient pyridox(am)ine 5′-phosphate oxidase activity. Experiments utilizing ^13^C_4_-pyridoxine confirmed lower pyridox(am)ine 5′-phosphate oxidase activity and revealed increased fractional turnovers of PLP and pyridoxal, indicating increased PLP hydrolysis to pyridoxal in PLPHP-deficient cells. This effect could be partly counteracted by inactivation of pyridoxal phosphatase. PLPHP deficiency had a distinct effect on mitochondrial PLP and PMP, suggesting impaired activity of mitochondrial transaminases. Moreover, in YBL036C-deficient yeast, PLP was depleted and PMP accumulated only with carbon sources requiring mitochondrial metabolism. Lactate and pyruvate accumulation along with the decrease of tricarboxylic acid cycle intermediates downstream of α-ketoglutarate suggested impaired mitochondrial oxidative metabolism in PLPHP-deficient HEK293 cells. We hypothesize that impaired activity of mitochondrial transaminases may contribute to this depletion. Taken together, our study provides new insights into the pathomechanisms of *PLPBP* deficiency and reinforces the link between PLPHP function, vitamin B_6_ metabolism, and mitochondrial oxidative metabolism.

Pyridoxal phosphate homeostasis protein (PLPHP, formerly known as proline synthetase cotranscribed homolog (bacterial) (PROSC) (1)) is a pyridoxal 5′-phosphate (PLP, active form of vitamin B_6_)–binding protein of unknown molecular function. Biallelic pathogenic variants in the *PLPBP* coding for PLPHP have been identified as a novel cause of early-onset vitamin B_6_–dependent epilepsy (OMIM ∗604436) (1, 2, 3, 4). Vitamin B_6_–dependent epilepsy refers to a group of genetic disorders that through various mechanisms lead to a decreased availability of cellular PLP, an important cofactor involved in many different enzyme reactions notably in amino acid and neurotransmitter metabolism. These disorders cause encephalopathy, neurodevelopmental delay, and seizures that can be treated with vitamin B_6_ (pyridoxine (PN) and/or PLP) (reviewed in (5)). In PLPHP-deficient patients, low PLP concentrations have been detected in cerebrospinal fluid (CSF) (1) and plasma (3) prior to vitamin B_6_ treatment. Altered concentrations of several amino acids, which at least to some extent could be explained by decreased activity of PLP-dependent enzymes, were reported in CSF and plasma before and after initiation of vitamin B_6_ treatment (1, 2, 6). Encephalopathy, white matter abnormalities, and microcephaly were reported in several patients (1, 3, 4, 6, 7, 8, 9). Elevated plasma lactate in pretreatment samples was often observed (1, 3, 4, 6). Seizures due to PLPHP deficiency are responsive to PN or PLP treatment (1, 2, 3, 4, 6, 7, 9, 10). For some patients, additional treatment with antiepileptic drugs (1, 2, 7) or folinic acid (3, 7) was required, while one reported patient had no seizures (3). Depending on the variant, the developmental outcome of patients varied from normal development (2) to a mild or severe neurodevelopmental delay (1, 2, 3) or even death if not treated timely with vitamin B_6_ (1, 3, 4). Plasma vitamin B_6_ profiles do not differentiate PLPHP patients from patients with other vitamin B_6_–dependent epilepsies and thus are insufficient for laboratory diagnosis in the absence of additional studies (2). No biomarkers specific to PLPHP deficiency are available, which may lead to delayed diagnosis and subsequent fatal outcome (4, 8).

PLP, the active form of vitamin B_6_, in humans is required for 44 (78 in *Metazoa*, 68 in *Fungi*) documented unique enzymatic reactions predominantly involved in amino acid and neurotransmitter metabolism (11) and is thus essential for normal brain development and function. Bacteria, fungi, and plants, but not humans, have at least one *de novo* PLP synthesis pathway (reviewed in (12)). In the yeast *Saccharomyces cerevisiae* (unicellular fungus), *de novo* PLP synthesis is catalyzed by PLP synthase, a bifunctional enzyme (Sno1 and Snz1 subunits) which uses L-glutamine, D-ribulose 5-phosphate (pentose phosphate pathway intermediate), and D-glyceraldehyde 3-phosphate (glycolytic intermediate) to yield L-glutamate and PLP (reviewed in (13)). The transcription of SNO1 and SNZ1 is activated in the late stationary phase (13). All above mentioned organisms can produce PLP *via* the salvage pathway from pyridoxal (PL) by pyridoxal kinase (PDXK) (BUD16 in yeast) or from PN and pyridoxamine (PM) *via* the consecutive action of PDXK and pyridox(am)ine phosphate oxidase (PNPO) (PDX3 in yeast) (Fig. 1) (12). The interconversion of PLP to pyridoxamine 5′-phosphate (PMP) also occurs during the catalytic cycle of transaminases (Fig. 1). PLP released during enzyme turnover is degraded by pyridoxal phosphatase (PDXP) to PL, which is rephosphorylated to PLP by PDXK (Fig. 1). Due to the reactivity of the 4′-aldehyde group, cellular PLP levels need to be tightly regulated to avoid potentially harmful nonspecific reactions while allowing sufficient PLP production to assure normal functioning of PLP-dependent enzymes. Some degree of regulation is achieved by product (PLP) inhibition of PDXK (14) and PNPO (15, 16, 17). Phosphatases, including PDXP, also contribute to the regulation of cellular PLP concentration (18, 19). It has been proposed that PLPHP is involved in the regulation of cellular PLP homeostasis as a PLP chaperone that protects newly synthesized PLP from degradation by phosphatases and nonspecific reactions and delivers PLP to apo-PLP enzymes (1, 20). While initially PLPHP was presumed to be a cytosolic protein (21), later on also mitochondrial localization was demonstrated (3, 22). The fact that some of the reported PLPHP-deficient patients presented with the symptoms of severe mitochondrial encephalopathy [3, 4] suggests that PLPHP may indeed have an important function in mitochondria. In support of this notion, the growth of YBL036C-deficient yeast has been shown to be strongly inhibited when grown on carbon sources requiring mitochondrial metabolism (3).

**Figure 1:**
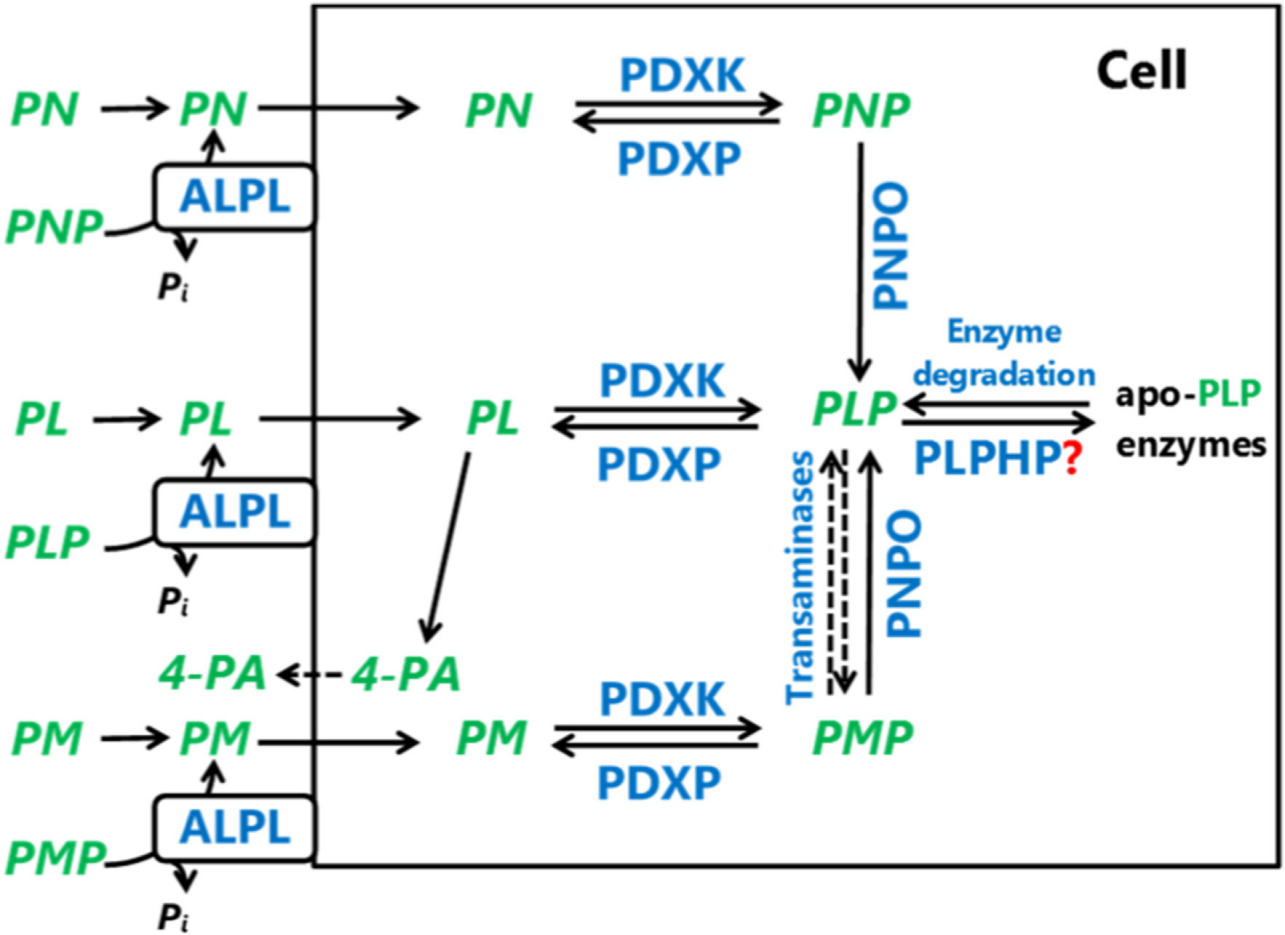
**Schematic representation of mammalian vitamin B**_**6**_**metabolism.** B_6_ vitamers are shown in *green*, enzymes are shown in *blue*. ALPL, tissue-nonspecific alkaline phosphatase; PA, 4-pyridoxic acid; PDXK, pyridoxal kinase; PDXP, pyridoxal phosphate phosphatase; PL, pyridoxal; PLP, pyridoxal 5′-phosphate; PLPHP, pyridoxal 5′-phosphate homeostasis protein; PN, pyridoxine; PNP, pyridoxine 5′-phosphate; PNPO, pyridoxamine 5′-phosphate oxidase; PM, pyridoxamine; PMP, pyridoxamine 5′-phosphate.

The aim of this study was to clarify molecular mechanisms underlying regulation of vitamin B_6_ metabolism by PLPHP and the link to mitochondrial dysfunction in PLPHP deficiency. To this end, we examined the interplay between PLPHP deficiency, vitamin B_6_, and organic acid and amino acid metabolism in human skin fibroblasts, human embryonic kidney cells (HEK293), and yeast *S. cerevisiae*.

## Results

### PLPHP deficiency leads to decreased cellular PLP level and accumulation of PNPO substrates

**Figure 2:**
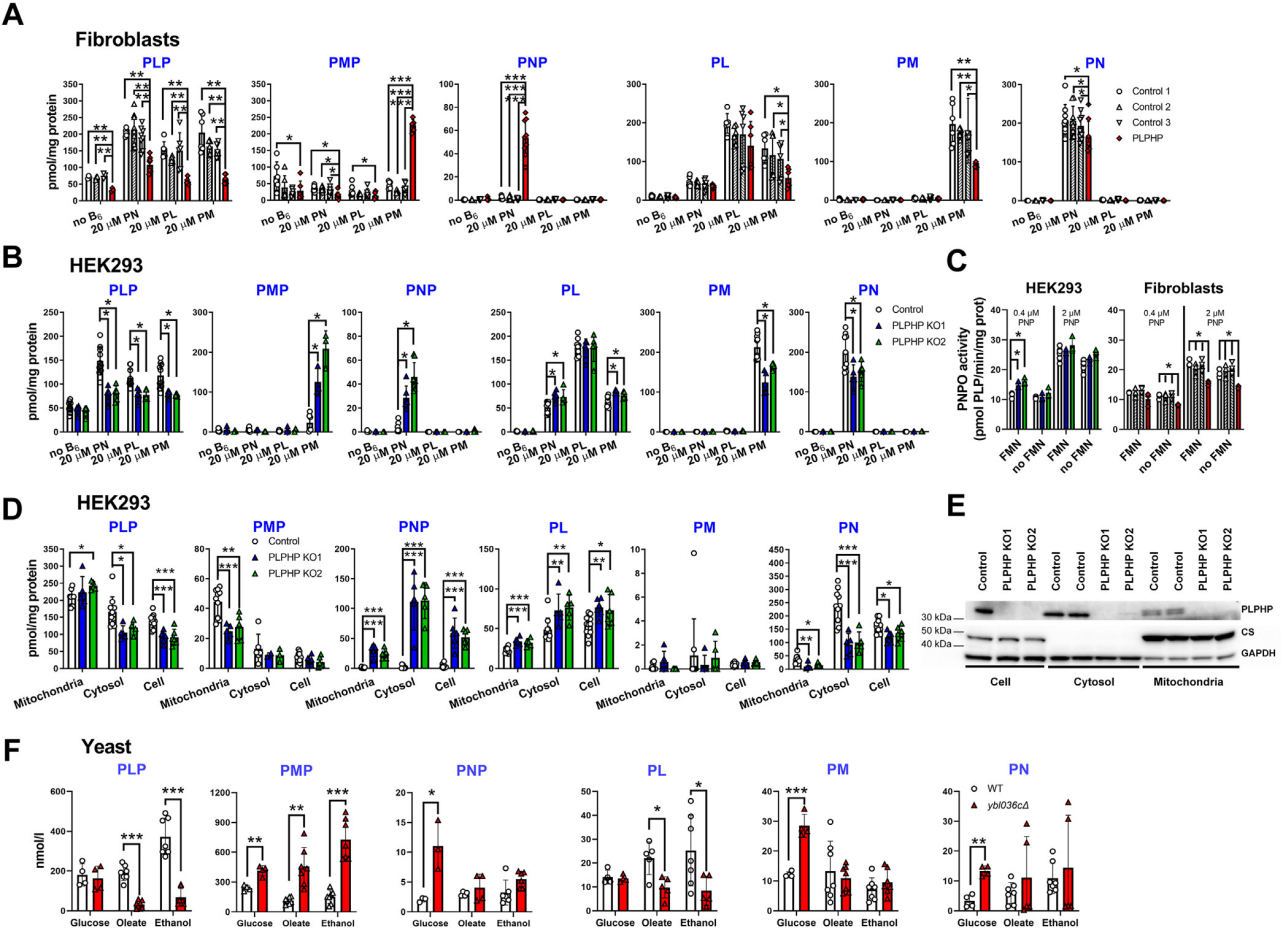
**The consequences of PLPHP deficiency on the intracellular vitamin B**_**6**_**metabolism in cultured human and yeast cells.***A*, intracellular content of PLP, PNP, PMP, pyridoxal (PL), pyridoxine (PN), and pyridoxamine (PM) in control and PLPHP-deficient fibroblasts cultured in complete DMEM containing no vitamin B_6_, 20 μM PN, 20 μM PL, or 20 μM PM for 96 h. The type of B_6_ vitamer present in the culture medium is indicated on the x-axis. Data are means from n = 6 to 9 biological replicates per cell line and condition, ±SD. ∗*p* < 0.05, ∗∗*p* < 0.01, and ∗∗∗*p* < 0.001 compared to control (only comparisons at the same culturing condition are shown). *B*, comparison of intracellular PLP, PNP, PMP, PL, PN, and PM content in control and PLPHP-deficient HEK293 cells cultured in complete DMEM containing no vitamin B_6_, 20 μM PN, 20 μM PL, or 20 μM PM for 96 h. The type of B_6_ vitamer present in the culture medium is indicated on the x-axis. Data are means from n = 6 to 12 biological replicates per cell line and condition, ±SD. ∗*p* < 0.05 compared to control (only comparisons at the same culturing condition are shown). *C*, PNPO enzyme activity with PNP as the substrate in control and PLPHP-deficient fibroblasts and HEK293 cells. PNPO activity was measured at subsaturating (0.4 μM) and saturating (2 μM) PNP concentrations. Data are means from n = 3 biological replicates per cell line and condition, ±SD. ∗*p* < 0.05 compared to control. *D*, B_6_ vitamer profiles in mitochondrial and cytosolic fractions, and total cell lysates in control and PLPHP-deficient HEK293 cells cultured with 19.4 μM pyridoxine (standard DMEM). Data are means from n = 5 to 6 biological replicates per cell line, ±SD. ∗*p* < 0.05, ∗∗*p* < 0.01, and ∗∗∗*p* < 0.001 compared to control. *E*, Western blot showing PLPHP protein expression in total cell extracts, and cytosolic and mitochondrial fractions in HEK293 cells cultured with pyridoxine (standard complete DMEM). *F*, intracellular B_6_ vitamer concentrations in WT and YBL036C-deficient (*ybl036cΔ*) yeast grown on glucose, oleate, or ethanol as carbon source. Data are means from n = 3 independent experiments, ±SD. ∗*p* < 0.05, ∗∗*p* < 0.01, and ∗∗∗*p* < 0.001 compared to WT yeast grown on the same carbon source. CS, citrate synthase; GAPDH, glyceraldehyde-3-phosphate dehydrogenase; PLP, pyridoxal 5′-phosphate; PLPHP, pyridoxal 5′-phosphate homeostasis protein; PMP, pyridoxamine 5′-phosphate; PNP, pyridoxine 5′-phosphate; PNPO, pyridox(am)ine 5′-phosphate oxidase.

The human diet contains a mixture of B_6_ vitamers. PN, PL, and PM can serve as the precursors for the synthesis of intracellular PLP (Fig. 1*A*). To obtain a detailed picture of how PLPHP deficiency affects vitamin B_6_ metabolism, we cultured PLPHP-deficient and control human skin fibroblasts and HEK293 cells in complete Dulbecco’s modified Eagle’s medium (DMEM) without vitamin B_6_ or with 20 μM PN, 20 μM PL, or 20 μM PM for 96 h and analyzed cellular B_6_ vitamer profiles. Independent of the type of B_6_ vitamer in the culture medium (PN, PL, or PM), PLP levels were significantly lower in PLPHP-deficient fibroblasts (Fig. 2*A*) and HEK293 cells (Fig. 2*B*) than control cells. Culturing cells without vitamin B_6_ led to strong reduction of PLP content in both control and PLPHP-deficient cells (Fig. 2, *A* and *B*). Under these culture conditions, PLP content was similar in PLPHP-deficient and control HEK293 cells, while PLP content in PLPHP-deficient fibroblasts remained lower than controls (Fig. 2, *A* and *B*). In PLPHP-deficient fibroblasts and HEK293 cells cultured in the presence of 20 μM PM, intracellular PM levels were lower while PMP was strongly increased compared to controls (Fig. 2, *A* and *B*). Culturing cells in the presence of 20 μM PN led to lower intracellular PN and increased pyridoxine 5′-phosphate (PNP) levels in PLPHP-deficient fibroblasts and HEK293 cells compared to controls (Fig. 2, *A* and *B*). Accumulation of PMP and PNP in cells cultured in the presence of PM and PN, respectively, suggested that PLPHP deficiency negatively affected PNPO activity. Since PNPO enzyme activity has been shown to be inhibited by its product PLP (15, 16, 17) and considering the proposed PLP chaperone function of PLPHP (1, 20), it is conceivable that PLPHP may stimulate PNPO activity by binding newly synthesized PLP, while PLPHP deficiency would inhibit PNPO activity. We did not detect co-immunoprecipitation of PLPHP in a PNPO IP in control HEK293 cells (Fig. S1), suggesting that if PLPHP regulates PNPO activity, the mechanism does not involve a physically stable interaction between the two proteins. PNPO enzyme activity measured in total cell lysates with subsaturating and saturating substrate concentration (0.4 μM and 2 μM PNP, respectively (Km for PNP 0.319 μM (23))) and endogenous FMN (PNPO cofactor) was ∼24% (*p* < 0.05) and 28% (*p* < 0.05) lower in PLPHP-deficient fibroblasts than that of controls, while in PLPHP-deficient HEK293 cells, PNPO activity was comparable to control cells (Fig. 2*C*). Addition of FMN in the assay mix led to the normalization of PNPO activity with subsaturating PNP concentration in PLPHP-deficient fibroblasts and an increase in PNPO activity in PLPHP-deficient HEK293 cells (Fig. 2*C*), suggesting that FMN may be limiting in PLPHP deficiency leading to insufficient PNPO activity and accumulation of PNPO substrates. However, FMN had no effect on PNPO activity assayed at saturating PNP concentration in both PLPHP-deficient fibroblasts and HEK293 cells. Moreover, supplementing riboflavin (precursor of FMN) in the cell culture medium for 96 h did not prevent PNP accumulation and had no effect on other B_6_ vitamers in PLPHP-deficient HEK293 cells and fibroblasts cultured with PN (Fig. S2).

Intracellular PL concentration was increased in PLPHP-deficient HEK293 cells cultured with PN or PM (Fig. 2*B*). Cellular concentration of PL degradation product 4-pyridoxic acid (4-PA) was independent of vitamin B_6_ status (Fig. S3, *A* and *B*).

### PLPHP deficiency affects mitochondrial vitamin B_6_ metabolism

It is assumed that the PLP salvage (Fig. 1) takes place in the cytosol. Mitochondria show virtually no, if any, PDXK and PNPO activity (24), but they take up PLP synthesized in the cytosol (25), which is required for the activity of mitochondrial transaminases. Transaminases catalyze the reversible transfer of an amino group from an amino acid (AA1) to an α-keto acid (KA1) forming a new amino acid (AA2) and α-keto acid (KA2) (AA1+KA1↔AA2+KA2). During the catalysis, PLP transiently accepts the amino group from AA1 forming PMP (AA1+PLP-enzyme↔KA2+PMP-enzyme), followed by transfer of this amino group to KA1 to form AA2 and regeneration of PLP (PMP-enzyme+KA1↔AA2+ PLP-enzyme). While the availability of PLP influences the activity of transaminases, it is clear from the reaction scheme above that the activity of transaminase itself influences PLP and PMP cellular content, depending on the substrate availability. For example, the availability of transaminase substrates glutamate and α-ketoglutarate (α-KG) have been shown to readily affect PLP and PMP concentrations in isolated rat liver mitochondria (24). Since PLPHP was shown to be present also in mitochondria (3, 22), we compared B_6_ vitamer profiles in mitochondrial and cytosolic fractions and total cell lysates of control and PLPHP-deficient HEK293 cells cultured with PN. In the cytosolic fraction, the changes in B_6_ vitamer concentrations caused by PLPHP deficiency followed the same pattern as in the total cell lysate (Fig. 2*D*). In contrast, in the mitochondrial fraction, PLP tended to be higher (lower in cytosolic fraction and total cell lysate), while PMP was strongly decreased (not significantly changed in the cytosolic fraction and total cell lysate) in PLPHP-deficient cells compared to controls (Fig. 2*D*). Since these cells were cultured with PN and assuming that there is no PNPO in mitochondria (24), the only source of mitochondrial PMP in this experiment would be different mitochondrial transaminases. Therefore, decreased mitochondrial PMP suggests that PLPHP deficiency affects the activity of mitochondrial transaminases, specifically the first half of transaminase reaction (see above). If we assume that regulation of transaminase activity is purely through metabolite availability, decreased PMP could be caused by shortage of AA1 or increased availability of KA2. However, to conclude with certainty, one should measure amino and keto acid concentrations in mitochondrial fractions. The effect of PLPHP deficiency on PL, PNP, and PN levels in the mitochondrial fraction followed a similar pattern as in the cytosolic fraction and total cell lysate (Fig. 2*D*). PDXP is present in the mitochondrial intermembrane space (24) explaining the presence of PL in mitochondrial fraction and possibly the similar patterns of PLPHP deficiency in the cytosolic and mitochondrial fractions. Since PNP and PN do not have a function in mitochondria because of the absence of PDXK and PNPO (24), PNP and PN detected in the mitochondrial fraction likely derive from contamination with the cytosolic fraction. While ∼7-fold depletion of PN (presumably exclusively cytosolic B_6_ vitamer) in the mitochondrial fraction compared to cytosolic fraction suggests quite good separation of cellular fractions, one should keep in mind that impurities in the fractions could lead to slight dilution of the observed effects. The GAPDH signal in the Western blot of the mitochondrial fraction (Fig. 2*E*) supports this notion. Western blot analysis of mitochondrial and cytosolic fractions showed that PLPHP protein was present in both fractions (Fig. 2*E*), confirming its previously reported mitochondrial localization (3, 22).

It was reported that the growth of YBL036C (PLPHP ortholog)-deficient yeast was strongly inhibited under conditions when grown on carbon sources requiring mitochondrial metabolism (3), which reinforces the notion that also in yeast, this protein has a function in mitochondria. To investigate how this growth defect relates to vitamin B_6_ metabolism, we measured B_6_ vitamer concentrations in WT and *ybl036cΔ* yeast grown on glucose (mitochondria-independent metabolism), oleate, and ethanol (both mitochondria-dependent metabolism) as in (3). Since samples were collected in the exponential growth phase, we assume that only PLP salvage pathway was active, while the contribution of the *de novo* PLP synthesis pathway, which is activated in the late stationary phase (13), was negligible if any. While WT yeast had comparable B_6_ vitamer concentrations with all three carbon sources, in *ybl036cΔ* yeast, B_6_ vitamer concentrations were affected in a carbon source–specific manner (Fig. 2*F*). Most notably, PLP levels were strongly decreased in *ybl036cΔ* yeast grown on oleate and ethanol (mitochondria-dependent) but not when grown on glucose (mitochondria-independent) (Fig. 2*F*), that is, PLP was low under conditions when growth was impaired (3). Other abnormalities in *ybl036cΔ* yeast grown on oleate and ethanol included decreased PL and increased PMP levels (Fig. 2*F*). In *ybl036cΔ* yeast grown on glucose PMP, PNP, PM, and PN were elevated compared to WT cells (Fig. 2*F*). It should be noted that yeast culture media contained a mixture of B_6_ vitamers with PN being predominant (Fig. S3*D*) making data interpretation less straight forward when compared to human cells cultured with a single B_6_ vitamer. In *ybl036cΔ* yeast grown on glucose, the increase in intracellular PNP and PMP levels can be attributed to an effect on the activity of PDX3 (PNPO ortholog) and transaminases, respectively, since culture medium contained predominantly PN, but no PM and PMP (Fig. S3*D*). In *ybl036cΔ* yeast grown on oleate and ethanol, the increase in PMP can be caused both by an effect on transaminases and on PDX3 (culture medium contained some PM and PMP) (Fig. S3*D*). Independent of the carbon source, 4-PA levels were similar in WT and *ybl036cΔ* yeast (Fig. S3*C*).

### Effect of vitamin B_6_ supplementation on B_6_ metabolism in PLPHP-deficient cells

**Figure 3:**
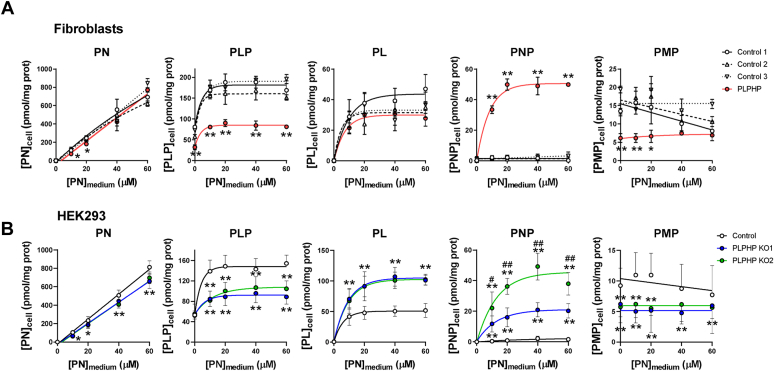
**Differential modulation oftheintracellular B**_**6**_**vitamer profilesby extracellular pyridoxine in control and PLPHP deficient cells.***A*, The dependence of the intracellular B_6_ vitamer concentrations on extracellular pyridoxine concentration in control and PLPHP-deficient fibroblasts. *B*, The dependence of the intracellular B_6_ vitamer concentrations on extracellular pyridoxine concentration in control and PLPHP-deficient HEK293 cells. Cells were cultured in complete DMEM containing varying concentrations of PN (0, 10, 20, 40, and 60 μM) for 96 h. B_6_ vitamer data were fit to a mono-exponential function in GraphPad Prism 8.3 (Y = Y0 + (Plateau-Y0)∗(1-exp(-K∗x)), shown as lines). Data are means from n = 9 to 18 biological replicates per cell line and condition, ±SD. ∗*p* < 0.05 and ∗∗*p* < 0.01 compared to each control (*A*). ∗*p* < 0.05 and ∗∗*p* < 0.01 compared to control; ^#^*p* < 0.05 and ^#^^#^*p* < 0.01 compared to PLPHP KO1 (*B*). All comparisons are at the same PN concentration (unpaired *t* test). PLPHP, pyridoxal 5′-phosphate homeostasis protein; PN, pyridoxine.

To determine how PLPHP deficiency affects cellular PLP homeostasis, we cultured control and PLPHP-deficient fibroblasts and HEK293 cells (Fig. 3, *A* and *B*, respectively) with varying concentrations of PN for 96 h and analyzed B_6_ vitamer profiles. While the cellular PN level increased with increasing extracellular PN concentration, the cellular PLP level reached saturation in both fibroblasts and HEK293 cells. In PLPHP-deficient cells, the PLP pool was saturated at a lower PLP concentration (Fig. 3, *A* and *B*, respectively). These data show that extracellularly added PN enters the cell, but it results in a blunted elevation of cellular PLP levels in PLPHP-deficient cells. Also for PL, saturation of the cellular pool was observed (Fig. 3). In agreement with data shown in Figure 2, *A* and *B*, cellular PL level in in PLPHP-deficient cells reached saturation at higher values than controls (Fig. 3*B*), while in PLPHP-deficient and control fibroblasts, there were no significant differences (Fig. 3*A*). Cellular PNP levels were very low and were not influenced by the extracellular PN concentration in control fibroblasts and control HEK293 cells (Fig. 3, *A* and *B*, respectively). In contrast, PNP levels increased with increasing PN concentration in PLPHP-deficient fibroblasts and HEK293 cells, suggesting that PNPO activity became more rate-limiting with increasing flux through the PLP synthesis pathway. Following the pattern observed for PLP and PL, PNP levels eventually reached saturation (Fig. 3, *A* and *B*, respectively). Intracellular PMP remained rather constant over the whole range of extracellular PN concentrations used and was about 50% lower in PLPHP-deficient cells at the different extracellular PN concentrations (Fig. 3).

### PLPHP deficiency primarily affects the protein-free PLP pool

**Figure 4:**
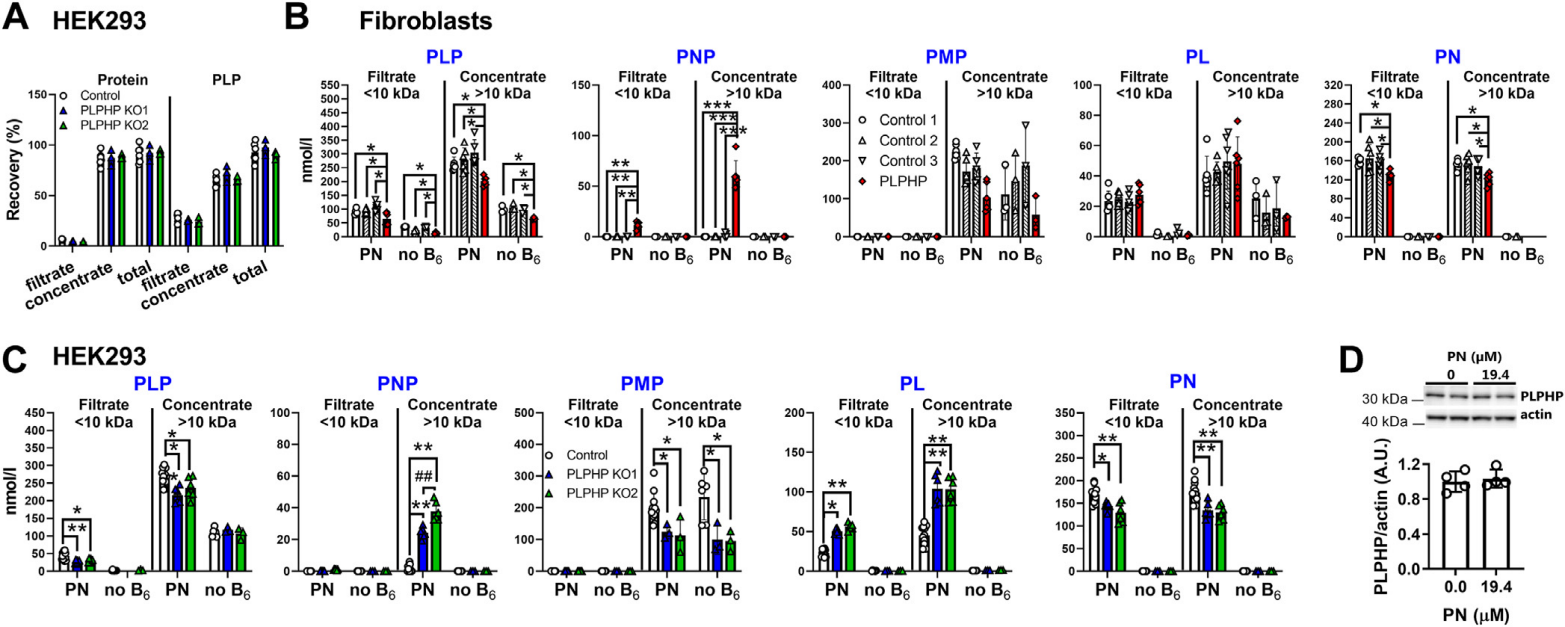
**The effects of PLPHP deficiency on protein-depleted and protein-enriched cell fractions.***A*, protein and PLP recovery after fractionation of HEK293 cells cultured with 19.4 μM PN for 96 h. *B*, B_6_ vitamer distribution between low-molecular weight (filtrate, relatively protein-free) and high-molecular weight (concentrate, protein-rich) fractions in control and PLPHP-deficient fibroblasts. *C*, B_6_ vitamer distribution between low- and high-molecular weight fractions in control and PLPHP-deficient HEK293 cells. *D*, Western blot showing PLPHP protein expression in control HEK293 cells cultured with no vitamin B_6_ or 19.4 μM PN for 96 h. Data are means from n = 3 to 6 (*A*); n = 6 (PN) and n = 3 (no B_6_) (*B*); n = 6 to 12 (PN) and n = 3 to 6 (no B_6_) (*C*); and n = 2 (*D*) per cell line and condition, ±SD. ∗*p* < 0.05, ∗∗∗*p* < 0.001, ^#^*p* < 0.05, and ^X^*p* < 0.05 compared to control 1, 2, and 3, respectively (*B*); ∗*p* < 0.05 and ∗∗*p* < 0.01 compared to control, and ^##^*p* < 0.01 compared to PLPHP KO1 (*C*). All comparisons are at the same culturing condition (one way ANOVA with Tuckey’s post hoc test). PLP, pyridoxal 5′-phosphate; PLPHP, pyridoxal 5′-phosphate homeostasis protein; PN, pyridoxine.

Next, we examined how PLPHP affects the distribution of PLP between protein-depleted and protein-enriched pools in human cells. Using centrifugal filters (cut-off 10 kDa), we fractionated total lysates of cells cultured without vitamin B_6_ or with 19.4 μM PN for 96 h and quantified B_6_ vitamers in the filtrate (protein-depleted) and concentrate (protein-enriched) (Fig. 4). The average molecular weight of human PLP-dependent enzymes listed in B_6_ database is 58.6 kDa (54.3, 34.6, and 112.7 kDa, median, minimum, and maximum, respectively) (11). Therefore, PLP covalently bound to enzymes is expected to remain in the concentrate, while protein-free (and bound to protein <10 kDa) B_6_ vitamers are expected to distribute at an equal concentration in the filtrate and concentrate. Protein recovery analysis after fractionation of HEK293 cells cultured in the presence of 19.4 μM PN showed that 92.5% of total protein was recovered, 87% in concentrate (>10 kDa) and 5.5% in filtrate (<10 kDa), with no differences between genotypes (Fig. 4*A*), showing that filtrates were indeed strongly protein-depleted. Under the same culture conditions, 94% of cellular PLP was recovered with 67% in the concentrate and 27% in the filtrate (Fig. 4*A*). B_6_ vitamer patterns in the filtrates and concentrates (Fig. 4, *B* and *C*) largely reflected B_6_ vitamer patterns seen in the total cell lysates (Fig. 2, *A* and *B*). Fractionation data suggested that the only truly free B_6_ vitamer was PN, while PMP was exclusively protein-bound (presumably to transaminases). PNP was also largely protein-bound, while PLP and PL existed in free and protein-bound forms in both fibroblasts and HEK293 cells (Fig. 4, *B* and *C*, respectively). PLP levels were lower in both the filtrate and concentrate in PLPHP-deficient and control fibroblasts and HEK293 cells cultured in the presence of 19.4 μM PN (Fig. 4, *B* and *C*). PLP concentrations in the filtrate of control fibroblasts were higher than in HEK293 cells, suggesting a larger protein-free PLP pool size in fibroblasts. Vitamin B_6_ starvation for 96 h led to almost complete depletion of PLP in the filtrates from control and PLPHP-deficient HEK293 cells (Fig. 4*C*), while in fibroblasts’ filtrates, PLP was strongly decreased, but not completely depleted, compared to the condition with 19.4 μM PN. PLP concentrations in the concentrates from PLPHP-deficient and control HEK293 cells after vitamin B_6_ starvation were similar, while in the concentrate from PLPHP-deficient fibroblasts, PLP was lower than controls. Despite clear differences in free PLP in control HEK293 cells cultured without vitamin B_6_ or 19.4 μM PN (Fig. 4*C*), PLPHP protein expression was similar in control HEK293 cells cultured without vitamin B_6_ or 19.4 μM PN (Fig. 4*D*). These data show that at least in this concentration range, PLPHP protein expression is not influenced by changes in the free PLP concentration. Taken together, these data show that when vitamin B_6_ supply to the cell is limited, both protein-bound and free PLP pools decrease, until the free pool is completely depleted. The remaining protein-bound PLP is likely bound to enzymes with long turnover half-lives. Under these conditions, PLPHP deficiency has either no effect (HEK293 cells) or less effect (fibroblasts) than under PLP-saturated conditions.

### PLPHP deficiency leads to increased PLP turnover in HEK293 cells and fibroblasts

**Figure 5:**
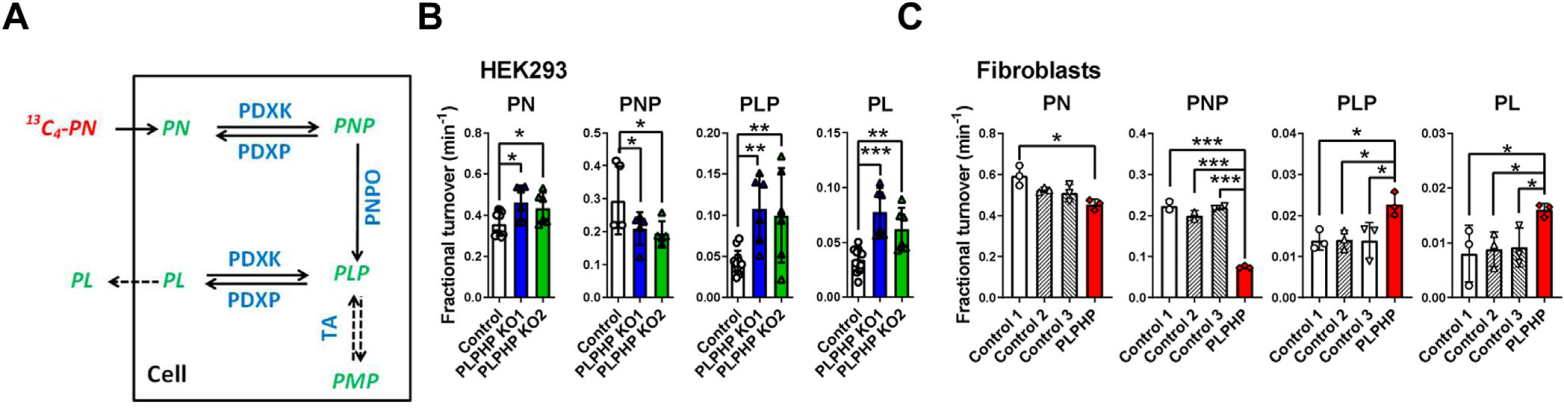
**PLPHP deficiency leads to increased fractional turnover of PLP.***A*, schematic representation of the vitamin B_6_ pathway upon supplementation with ^13^C_4_-pyridoxine (^13^C_4_-PN). Abbreviations as in Figure 1*A*. *B*, fractional turnovers of pyridoxine (PN), pyridoxine 5′-phosphate (PNP), pyridoxal 5′-phosphate (PLP), and pyridoxal (PL) in control and PLPHP-deficient HEK293 cells calculated from the time courses of fractional enrichments of ^13^C_4_-PN, ^13^C_4_-PNP, ^13^C_4_-PLP, and ^13^C_4_-PL (Fig. S5*D*). Data are means from n = 6 to 12 per cell line, ±SD. ∗*p* < 0.05, ∗∗*p* < 0.01, and ∗∗∗*p* < 0.001 compared to control (one way ANOVA with Tuckey’s post hoc test). *C*, fractional turnovers of PN, pyridoxine 5′-phosphate (PNP), pyridoxal 5′-phosphate (PLP), and PL in control and PLPHP-deficient fibroblasts calculated from the time courses of fractional enrichments of ^13^C_4_-PN, ^13^C_4_-PNP, ^13^C_4_-PLP, and ^13^C_4_-PL (Fig. S5*B*). Data are means from n = 3 per cell line, ±SD. ∗*p* < 0.05 and ∗∗∗*p* < 0.001 compared to control (one way ANOVA with Tuckey’s post hoc test). PLPHP, pyridoxal 5'-phosphate homeostasis protein; TA, transaminase.

To determine how PLPHP deficiency affects the dynamics of vitamin B_6_ metabolism, we supplemented cells with ^13^C_4_-labeled PN (^13^C_4_-PN) in otherwise vitamin B_6_–free culture medium (Fig. 5*A*) and followed the incorporation of the label into the intracellular PN, PNP, PLP, and PL pools (Fig. S4, *A* and *C*). In agreement with the steady-state B_6_ vitamer quantification described above, the production of ^13^C_4_-PNP and ^13^C_4_-PL was increased, while that of ^13^C_4_-PLP was decreased in both PLPHP-deficient fibroblasts and HEK293 cells (Fig. S4, *A* and *C*). Of note, no ^13^C_4_ labeling of PMP was observed during the duration of the experiment (1 h), suggesting that the observed dynamics likely occur in protein-free B_6_ vitamer pools, since under these experimental conditions, PMP appears to be exclusively protein-bound (Fig. 4). From the fractional enrichment time courses (Fig. S4, *B* and *D*, fibroblasts and HEK293 cells, respectively), we calculated the fractional turnover rates (fraction of total pool replaced per unit of time) of PN, PNP, PLP, and PL. Fractional turnovers rates of PN, PLP, and PL were increased, while the fractional turnover of PNP was decreased in PLPHP-deficient HEK293 cells (Fig. 5*B*), indicating limitation of PNPO activity. The changes in turnover rates in PLPHP-deficient fibroblasts were comparable to those observed in HEK293 cells, with exception of fractional turnover of PN, which was decreased (Fig. 5*C*). Increased fractional turnovers of PLP and PL suggested increased PLP hydrolysis to PL in PLPHP-deficient cells. In agreement, measurement of PL appearance in the culture medium of HEK293 cells cultured with PN showed increased secretion of PL in PLPHP-deficient cells compared to control cells (Fig. S5).

### Decreasing PDXP activity restores cellular PLP concentration in PLPHP-deficient HEK293 cells

**Figure 6:**
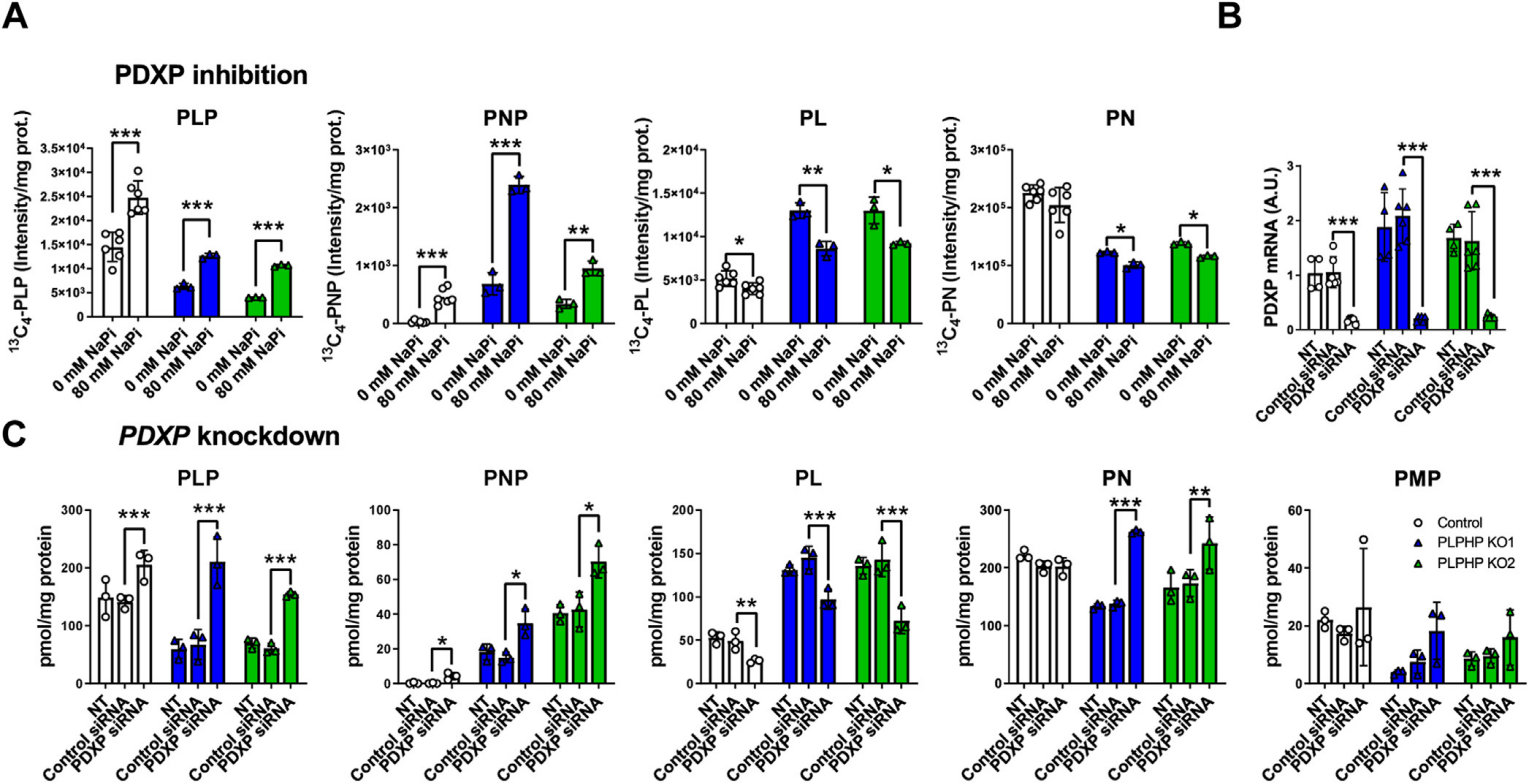
**Decreasing PDXP activity restores cellular PLP content in PLPHP-deficient HEK293 cells.***A*, the effect of nonspecific PDXP inhibition with 80 mM sodium phosphate (NaPi) for 60 min on the content of ^13^C_4_-labeled B_6_ vitamers in control and PLPHP-deficient HEK293 cells. Data are means from n = 3 per cell line and condition, ±SD. ∗*p* < 0.05, ∗∗*p* < 0.01, and ∗∗∗*p* < 0.001 compared to 0 mM NaPi at the same genotype (unpaired *t* test). *B*, the effect of *PDXP* knockdown with siRNA for 48 h on *PDXP* mRNA levels in control and PLPHP-deficient HEK293 cells. Data are means from n = 3 per cell line and condition, ±SD. ∗∗∗*p* < 0.001 compared to control siRNA at the same genotype (unpaired *t* test). *C*, the effect of *PDXP* knockdown with siRNA for 48 h on B_6_ vitamer content in control and PLPHP-deficient HEK293 cells. Data are means from n = 3 per cell line and condition, ±SD. ∗*p* < 0.05, ∗∗*p* < 0.01, and ∗∗∗*p* < 0.001 compared to control siRNA at the same genotype (unpaired *t* test). NT, not transfected. PDXP, pyridoxal phosphatase; PLP, pyridoxal 5'-phosphate; PLPHP, pyridoxal 5′-phosphate homeostasis protein.

Since experiments with ^13^C_4_-PN showed increased PLP and PL turnover in both PLPHP-deficient HEK293 cells and fibroblasts, we tested whether blocking PLP hydrolysis to PL can restore cellular PLP. PDXP can be inhibited with a high concentration of inorganic phosphate (18). Therefore, we incubated control and PLPHP-deficient HEK293 cells with 80 mM NaPi as described in (18) and 20 μM ^13^C_4_-PN for 60 min and quantified ^13^C_4_ incorporation in PN, PNP, PLP, and PL (Fig. 6*A*). Nonspecific inhibition of cellular phosphatases led to an increase in ^13^C_4_-PLP and a decrease in ^13^C_4_-PL, with a concomitant increase in ^13^C_4_-PNP and decrease in ^13^C_4_-PN (Fig. 6*A*). *PDXP* knockdown using siRNA for 48 h (Fig. 6*B*) had an effect comparable to PDXP inhibition, that is, cellular PLP concentration was increased to values similar to the control cells, PL concentration decreased, and both PNP and PN accumulated (Fig. 6*C*). Accumulation of PNP in response to PDXP inhibition and knockdown was likely caused by a combination of product (*i.e.*, PLP) inhibition of PNPO (15, 16) and decreased hydrolysis of PNP by PDXP.

### The interplay between vitamin B_6_ metabolism, organic and amino acid metabolism, and mitochondrial oxidative function

**Figure 7:**
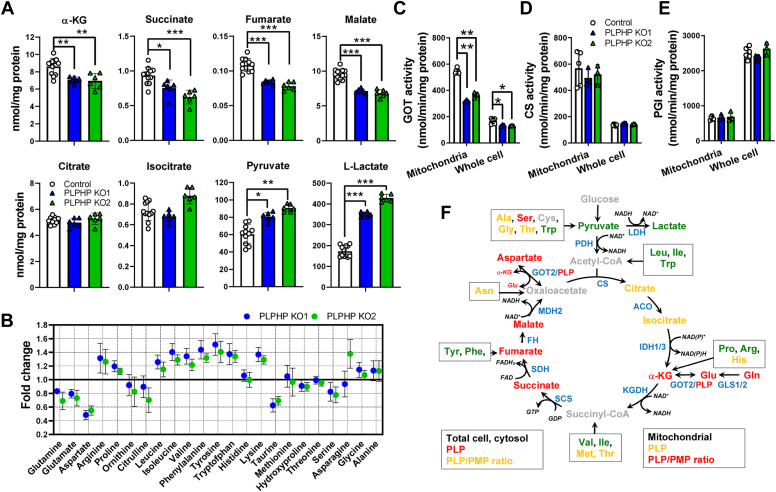
**Metabolic alterations in response to PLPHP deficiency in human HEK293 cells suggest mitochondrial dysfunction.***A*, organic acid concentrations in control and PLPHP-deficient HEK293 cells. Cells were cultured in standard DMEM containing 19.4 μM pyridoxine. Data are means from n = 6 to 12 per cell line, ±SD. ∗*p* < 0.05, ∗∗*p* < 0.01, ∗∗∗*p* < 0.001 compared to control HEK293 cells. *B*, alterations in amino acid profiles in response to PLPHP deficiency in HEK293 cells. Cell culture conditions as in panel *A*. Data are from n = 6 to 12 per cell line, ±SD. Data are shown as fold change in amino acid concentration PLPHP KO/Control. Actual amino acid concentrations are shown in Fig. S7. *C*, the activity of glutamic-oxaloacetic transaminase (GOT) in mitochondrial fractions and total cell lysates of control and PLPHP-deficient HEK293 cells. Cell culture conditions as in panel *A*. Data are means from n = 6 to 12 per cell line, ±SD. ∗*p* < 0.05 and ∗∗*p* < 0.01 compared to control HEK293 cells. *D*, the activity of mitochondrial marker enzyme citrate synthase (CS) in mitochondrial fractions and total cell lysates of control and PLPHP-deficient HEK293 cells. Cell culture conditions as in panel A. Data are means from n = 6 to 12 per cell line, ±SD. *E*, the activity of cytosolic marker enzyme phosphoglucoisomerase (PGI) in mitochondrial fractions and total cell lysates of control and PLPHP-deficient HEK293 cells. Cell culture conditions as in panel *A*. Data are means from n = 6 to 12 per cell line, ±SD. *F*, schematic summary of consequences of PLPHP deficiency on organic and amino acid metabolism in HEK293 cells cultured with pyridoxine. PLPHP deficiency leads to depletion of TCA cycle intermediates downstream of IDH1/3 (possibly due to decreased anaplerosis from amino acids) and decreased pyruvate oxidation and lactate accumulation. *Red* – decreased, *green* – increased, *yellow* – not changed, *gray* – not measured metabolites. Enzymes are depicted in *blue*. ACO, aconitase; CS, citrate synthase; FH, fumarate hydratase; GOT2, glutamic-oxaloacetic transaminase 2; IDH1/3, isocitrate dehydrogenase 1 and 3; KGDH, α-ketoglutarate dehydrogenase; LDH, lactate dehydrogenase; MDH, malate dehydrogenase; PDH, pyruvate dehydrogenase; PLP, pyridoxal 5′-phosphate; PLPHP, pyridoxal 5′-phosphate homeostasis protein; PMP, pyridoxamine 5′-phosphate; SCS, succinyl-CoA synthetase; SDH, succinate dehydrogenase.

Next, we investigated broader metabolic consequences of PLPHP deficiency. Data from *Salmonella enterica* Yggs (PLPHP ortholog) mutants suggested that perturbations in α-keto acid metabolism are tightly linked to Yggs function (26, 27). It was hypothesized that Yggs may regulate transamination reactions through an effect on α-keto acid concentration, such as α-KG, thereby affecting PLP and PMP concentrations (26, 27). The glutamate/α-KG couple participates in many reactions catalyzed by transaminases (28). α-KG is also an intermediate in the tricarboxylic acid (TCA) cycle and thus plays an important role in linking TCA cycle function with amino acid metabolism. To investigate the link between PLPHP function and α-KG concentration proposed in (26, 27), we quantified intracellular organic acid levels. α-KG level was 1.2-fold lower in PLPHP-deficient HEK293 cells (Fig. 7, *A* and *F*). In addition, several organic acids downstream of α-KG were decreased including succinate, fumarate, and malate, whereas citrate and isocitrate concentrations were not affected (Fig. 7, *A* and *F*). This could point to the insufficient synthesis of α-KG from glutamate, for example, due to impaired activity of GOT. Enzyme activity measurements revealed slightly lower total GOT activity (cytosolic GOT1 plus mitochondrial GOT2) in total cell lysates and more profoundly decreased GOT activity in mitochondrial fraction of PLPHP-deficient HEK293 cells (Fig. 7*C*). The similar pattern of GOT (Fig. 7*C*) and citrate synthase (CS, mitochondrial enzyme) activities (Fig. 7*D*) in contrast to phosphoglucose isomerase (PGI, cytosolic enzyme) (Fig. 7*E*) suggest that at least in HEK293 cells, mitochondria are the main contributors to the total cellular GOT activity. Increased pyruvate and lactate concentrations in PLPHP-deficient HEK293 cells indicated impaired mitochondrial pyruvate oxidation (Fig. 7, *A* and *F*).

Since many enzymes involved in amino acid metabolism use PLP as a cofactor, we analyzed amino acid profiles of PLPHP-deficient and control HEK293 cells (Figs. 7*B* and S6). The concentration of glutamine, glutamate, aspartate, ornithine, citrulline, taurine, and serine were lower, while arginine, proline, leucine, isoleucine, valine, phenylalanine, tyrosine, tryptophan, and lysine were increased in PLPHP-deficient compared to control HEK293 cells (Figs. 7*B* and S6). It is likely that the lower glutamine and glutamate levels contributed to the decrease in α-KG described above (Fig. 7, *A* and *F*). The accumulation of branched chain and aromatic amino acids is in agreement with the postulated effect of PLPHP deficiency on the activity of transaminases and may contribute to decreased replenishment of TCA cycle intermediates as summarized in Figure 7*F*. The lower aspartate concentration in PLPHP-deficient HEK293 also points towards a lower activity of GOT2, since GOT2 is the predominant enzyme generating aspartate in the mitochondrion followed by export to the cytosol (29) (Fig. 7*F*).

## Discussion

*PLPBP* deficiency, first described by Darin *et al.* (1) as *PROSC* deficiency, expanded the group of vitamin B_6_–dependent epilepsies. While the number of identified affected patients has increased to 57 (1, 2, 3, 4, 6, 7, 8, 9, 10, 30, 31, 32, 33, 34, 35, 36), the precise molecular function of PLPHP and how it regulates cellular PLP homeostasis is still unknown. To address this gap of knowledge, we performed a detailed analysis of consequences of PLPHP deficiency on vitamin B_6_ metabolism in human fibroblasts and HEK293 cells. We showed that independent of the type of extracellular B_6_ vitamer (PN, PM, or PL), intracellular PLP concentration was lower in PLPHP-deficient fibroblasts and HEK293 cells than controls. Both free and bound PLP was decreased in PLPHP-deficient fibroblasts and HEK293 cells. Furthermore, hydrolysis of PLP to PL was increased, which could be counteracted by inhibition or knockdown of PDXP. We also showed that under conditions of a high supply of extracellular vitamin B_6_ (PN or PM), PNPO activity became limiting in PLPHP-deficient cells, leading to accumulation of PNPO substrates PNP and PMP. B_6_ vitamer analysis in cytosolic and mitochondrial fractions of control and PLPHP-deficient HEK293 cells suggested an effect on mitochondrial transaminases, which was supported by lower GOT activity in mitochondrial fraction. Mitochondrial pyruvate oxidation was impaired as indicated by higher pyruvate and lactate levels in PLPHP-deficient HEK293 cells. This was accompanied by reduction of TCA cycle intermediates downstream of α-KG, possibly due to the decreased formation of TCA cycle intermediates from amino acids due to the impaired activity of PLP-dependent transaminases notably GOT2 (summarized in Fig. 7*F*). B_6_ vitamer analysis in WT and YBL036C-deficient yeast showed that PLP was strongly decreased only with carbon sources requiring mitochondrial metabolism. These data support the link between YBL036C function, vitamin B_6_ metabolism, and mitochondrial metabolism.

Untreated *PLPBP* deficiency patients show symptoms of PLP deficiency, including a low PLP concentration in CSF (1) and plasma (3) and seizures responsive to treatment with PN or PLP (reviewed in (7)). So, it seems quite unambiguous that in humans *in vivo*, PLPHP affects the intracellular availability of PLP. While there are no data on intracellular PLP concentration in tissues of patients, PLP was reported to be lower in *plpbp*^*−/−*^ zebrafish, the only animal model of this disease so far (3). In cell cultures, findings have been contradictory: elevated PLP in PLPHP-deficient patient fibroblasts (1), decreased PLP in PLPHP-deficient patient fibroblast and HEK293 cells (3), and unaltered intracellular PLP concentrations in *Escherichia coli* and *S. enterica yggS* mutants compared to the WT cells (26, 37, 38), while PLP secretion by *S. enterica yggS* mutant was strongly increased (26). These divergent findings may well be explained by the fact that different authors used different cell culture conditions which affect vitamin B_6_ homeostasis greatly especially in cell models (B_6_ vitamer type and concentration but possibly also other components of culture media). Several scenarios of how PLPHP could influence intracellular PLP are possible, including regulation of the following: (1) B_6_ vitamer uptake into the cell, (2) synthesis of PLP (PDXK and PNPO), (3) degradation of PLP by phosphatases (*i.e.*, PDXP), (4) binding of free PLP (PLP chaperone function) (1, 20), (5) activity of transaminases (26, 27), and (6) concentration of small PLP-reactive metabolites (similar to ALDH7A1 and ALDH4A1 deficiencies) (39, 40). Based on our observation that the uptake of ^13^C_4_-labeled PN was similar in WT and PLPHP-deficient fibroblasts and HEK293 cells (Fig. S4), it is not likely that PLPHP regulates cellular B_6_ vitamer uptake. Regarding regulation of PLP synthesis, there is no indication of reduced PDXK activity, since there is no accumulation of PL (substrate of PDXK) in PLPHP-deficient fibroblasts and HEK293 grown with only PL in the culture medium. Both steady state B_6_ vitamer analyses and dynamic experiments with ^13^C_4_-labeled PN revealed the accumulation of the PNPO substrates PNP and PMP (when PM was added to the culture medium), suggesting that PLPHP deficiency negatively affects PNPO activity in intact fibroblasts and HEK293 cells. However, the impairment of PNPO activity in intact cells appears to be relevant only at high extracellular B_6_ vitamer concentrations, which presumably results in high flux through the PLP synthesis pathway (PDXK/PNPO). We showed that PNPO activity measured in total cell lysates at both subsaturating and saturating PNP concentrations was lower in PLPHP-deficient fibroblasts but near normal in PLPHP-deficient HEK293 cells. Since PNPO activity was measured in total cell extract, it is not possible to determine the mechanistic cause of lower PNPO activity in PLPHP-deficient fibroblasts and cell type–specific differences. Studies with purified enzyme could provide more information about changes in enzyme kinetics and the role of FMN cofactor. The lack of effect on PNPO activity in PLPHP-deficient HEK203 cell lysates as opposed to impaired PNPO activity in living cells suggests that the regulation of PNPO activity by PLPHP deficiency requires either intact cell structures (which are lost upon preparation of cell lysates) or intact metabolic pathway activity. In literature, both increased (3) and unaltered (1) intracellular PNP levels were reported in PLPHP-deficient human cells, while in bacterial cells, mostly increased PNP was reported (1, 26, 37, 38). Our data indicate that these differences likely stem from variations in B_6_ vitamer concentrations in culture media used in the experiments. It is not clear how relevant limitation of PNPO activity by PLPHP deficiency is *in vivo*, when cells function under conditions of subsaturating B_6_ vitamer supply. For example, no PNP or PMP accumulation was reported in *plpbp*^*−/−*^ zebrafish receiving standard nutrition (3). The cause of the limitation of PNPO activity in PLPHP-deficient cells remains to be identified. One may speculate that PLPHP regulates PNPO activity, for example, by accepting newly synthesized PLP and relieving product inhibition of PNPO. In this scenario, the absence of PLPHP would result in decreased PNPO activity. However, if the sole function of PLPHP would be regulation of PNPO activity, PLPHP deficiency would affect cellular PLP concentration only under conditions requiring PNPO activity, that is, with PN and PM but not PL in the culture medium. However, we showed that cellular PLP concentration was lower in PLPHP-deficient fibroblasts and HEK293 cells cultured with PL, which suggests that the effect on PNPO activity is one but not the sole consequence of PLPHP deficiency. The sensitivity of PNPO enzyme activity assay to addition of FMN suggested that the availability of FMN cofactor could have been reduced in PLPHP deficiency.

With regard to the third scenario, in which PLPHP could influence degradation of PLP by phosphatases (*e.g.*, PDXP), we showed increased formation of ^13^C_4_-labeled PL in PLPHP-deficient fibroblasts and HEK293 cells supplemented with ^13^C_4_-labeled PN (Fig. S4). Moreover, inhibition and knockdown of PDXP in HEK293 cells resulted in normalization of cellular PLP and decreased PL content, indicating that indeed under these culture conditions, PLP is prone to degradation by PDXP in PLPHP-deficient cells. It has been shown that treatment of PLPHP-deficient patients with vitamin B_6_ led to strong increase of not only PLP, but also of PL and 4-PA in plasma (2, 3) with likely PDXP and other phosphatases contributing to the elevation of PL. Increased susceptibility of PLP for degradation by PDXP or other phosphatases in PLPHP-deficient cells is in line with a PLP-binding/chaperone function of PLPHP that regulates concentration of free PLP (1, 20). Both PLP-producing enzymes PDXK (14) and PNPO (15, 16) are product-inhibited. Assuming that PLPHP is indeed a PLP chaperone, in its absence newly synthesized PLP, if released from PDXK and PNPO, may indeed be more accessible to PDXP. Our data show that PLPHP protein expression itself is not regulated by changes in cellular (total, free, and bound) PLP concentration in control HEK293 cells.

Finally, PLPHP could influence cellular PLP concentration through an effect on the levels of small PLP-reactive metabolites. Such a negative effect on cellular PLP has been described for ALDH7A1 and ALDH4A1 (enzymes in lysine and proline metabolism, respectively) deficiencies (39, 40). However, such a scenario would only be possible if PLPHP had some enzymatic function. The reported cotranscription of bacterial *PLPBP* orthologs with *proC* coding for pyrroline-5-carboxylate reductase suggested that it may have an enzymatic function in proline metabolism (41, 42). The recent data on the capacity of human PLPHP to dimerize (43, 44) and the fact that yeast oligomeric Ybl036C has been shown to have enzymatic activity (45) strengthen the possibility that PLPHP does have an enzymatic function, which remains to be resolved in the future.

In agreement with previous reports (3, 22), we showed in HEK293 cells that PLPHP protein is expressed in both cytosol and mitochondria. The observation that some of the reported *PLPBP* deficiency patients presented with signs and symptoms mimicking those described in patients suffering from severe mitochondrial encephalopathy, causing delayed diagnosis and fatal outcome [3, 4], suggests that PLPHP is important for normal mitochondrial function. Lack of abnormalities in respiratory chain enzyme activities and cellular respiratory capacity in cultured PLPHP-deficient patient fibroblasts suggested that it was not an intrinsic defect in the oxidative phosphorylation pathway enzymes [3]. It has been shown that the growth of YBL036C-deficient yeast was strongly impaired specifically when grown on carbon sources requiring mitochondrial metabolism [3]. We showed profound abnormalities in B_6_ vitamer profiles in YBL036C-deficient yeast growing on carbon sources requiring mitochondrial metabolism. These abnormalities were not seen under growth conditions not requiring mitochondrial metabolism. Furthermore, we showed that PLPHP deficiency has a distinct effect on the mitochondrial PLP and PMP concentrations as compared to the cytosolic compartment in HEK293 cells. Based on the notion that there is no PDXK and PNPO in mitochondria, the alterations in mitochondrial PLP and PMP in response to PLPHP deficiency could only be brought about *via* an effect on mitochondrial transaminases. The fact that mitochondrial PLP was not decreased in PLPHP-deficient cells suggested that the activity of transaminases was affected not due to the shortage of the enzyme cofactor PLP. It rather hinted to the regulation by substrate(s) availability for transaminases. Such regulation, specifically *via* an effect on α-keto acid concentration, has been already suggested based on the observations in *S. enterica* Yggs mutants (26, 27). Such regulation mechanism would require that PLPHP would be involved in the metabolism of the putative substrate(s) of transaminases, reiterating the possibility of an enzymatic function of PLPHP. We showed that α-KG and TCA cycle intermediates downstream of α-KG were decreased, which could at least in part be attributed to the impaired activity of involved transaminases (*e.g.*, GOT2) in PLPHP-deficient cells. Figure 7*F* summarizes our observations and provides a mechanism of how PLPHP deficiency could impair mitochondrial oxidative metabolism *via* an effect on metabolite levels.

Taken together, we showed that in human cells, PLPHP deficiency had a complex, multifaceted effect on cellular vitamin B_6_ metabolism. Specifically, in PLPHP-deficient cells cultured under saturated vitamin B_6_ conditions, cellular PLP is decreased, PNPO activity is impaired, and PLP hydrolysis is increased. These effects could to some extent be explained by the PLP-chaperone function of PLPHP. Furthermore, our data provided hints on the mechanism which may link the impaired metabolism of vitamin B_6_ in PLPHP deficiency to the impairment in mitochondrial oxidative metabolism, which may contribute to a better understanding of the pathophysiology in *PLPBP* deficiency patients.

## Experimental procedures

### Human cell lines and culture conditions

Primary skin fibroblast cell lines of three controls and one PLPHP-deficient patient (patient 5 in (3), homozygous for 370–373del) were used. Written informed consent was obtained for all subjects. Two PLPHP-deficient HEK293 cells lines, generated using CRISPR/Cas9 (PLPHP KO1 (c.124_127delCTAG) and PLPHP KO2 (c.128_129ins131bp)), and two control clonal cell lines as described (3) were used.

Cells were routinely maintained in complete DMEM (#31966-021 (Gibco), containing 19.4 μM PN, 25 mM glucose, 1 mM pyruvate, 4 mM L-alanyl-L-glutamine) supplemented with 10% heat-inactivated fetal bovine serum (FBS) (#10270-106, Gibco) and 1% penicillin-streptomycin (PS) (#15140-122, Gibco) in a humidified atmosphere of 5% CO_2_ at 37 °C. Medium was refreshed every 48 h and 24 h prior to harvesting cells. To determine the effects of specific B_6_ vitamers in culture medium on cellular B_6_ vitamer profiles, custom made complete DMEM (like #31966 (contains no PN), Gibco) supplemented with varying concentrations of PN (Sigma-Aldrich), 20 μM PL HCl (Sigma-Aldrich), or 20 μM PM di-HCl (Sigma-Aldrich) was used.

### Yeast strains, culture conditions, and B_6_ vitamer extraction

The following *S. cerevisiae* strains were used: BY4742 (MATα his3Δ1 leu2Δ0 lys2Δ0 ura3Δ0) as the WT strain and knock-out strain *ybl036cΔ* (YBL036c::KAN) (Euroscarf) (3). All strains were grown in minimal medium containing 6.7 g/l BD Difco yeast nitrogen base without amino acids (#291940, Thermo Fisher Scientific), supplemented with 5 g/l glucose and amino acids (20 mg/l) for at least 24 h. Then cells were shifted to medium containing yeast nitrogen base, 25 mM potassium phosphate buffer (pH 6.0), and either 5 g/l glucose or 2 g/l oleate and 1 g/l yeast extract or 2 g/l ethanol and 1 g/l yeast extract as in (3). After culturing overnight, the optical density (OD) of the cultures was measured and cells were pelleted to OD = 2. Cell pellets were washed two times with PBS and stored as pellet (OD = 2) at −80 °C until further analysis. For B_6_ vitamer extraction, 300 μl of trichloroacetic acid (50 g/l) was added to the pellet (OD = 2). Cells were disrupted using glass beads (200 μl) for 5 min at 4 °C. Cell extracts were centrifuged at 2000 rpm for 5 min at 4 °C, and supernatants were stored at −80 °C until B_6_ vitamer analysis as described below.

### Preparation of mitochondrial and cytosolic fractions

HEK293 cells were cultured in 75 cm^2^ flasks in complete DMEM (#31966-021, Gibco) plus 10% FBS and 1% PS to confluency. Mitochondrial and cytosolic fractions were prepared as described (46) with small modifications. Briefly, cells were washed twice with 10 ml cold Dulbecco's phosphate-buffered saline (DPBS, BioWhittaker). Next, 10 ml of cold medium containing 100 mM sucrose, 1 mM EGTA, and 20 mM Mops (pH 7.4) were added, and cells were scraped with a disposable plastic scraper on ice and centrifuged for 5 min at 800*g*. Supernatant was aspirated, 1 ml of medium containing 100 mM sucrose, 1 mM EGTA and 20 mM Mops (pH 7.4), 10 mM triethanolamine, 5% Percoll, and 0.1 mg/ml digitonin was added, and cells were resuspended by pipetting and transferred into glass tissue grinder (2 ml) and incubated for 3 min on ice. Cells were homogenized with seven up-and-down strokes with a tight-fitting Teflon pestle mounted on a motor overhead at 500 rpm. Homogenate was centrifuged for 5 min at 800*g* at 4 °C. Supernatant was transferred to a new 1.5 ml eppendorf tube and centrifuged for 10 min at 10,000*g* at 4 °C. Supernatant (cytosolic fraction) was transferred to a new 1.5 ml eppendorf tube, and the pellet (mitochondrial fraction) was resuspended in 100 μl ultrapure H_2_O (for B_6_ vitamer analysis) or 100 μl DPBS (for enzyme activity assays and Western blotting). For B_6_ vitamer analysis, cytosolic and mitochondrial fractions were extracted with equal volume of trichloroacetic acid (50 g/l), vortexed, and centrifuged at 16,200*g* for 5 min at 4 °C. Eighty microliters of the supernatant were analyzed as described in section ‘B6 vitamer analysis’.

### Sample preparation for B_6_ vitamer analysis

For B_6_ vitamer analysis in total cell extracts, cells were cultured in 6-well plates in complete DMEM (like #31966 (contains no PN) (Gibco), 10% FBS, 1% PS) containing no vitamin B_6_, 20 μM PN, 20 μM PL, or 20 μM PL for 96 h to confluency. For the analysis of the dependence of cellular B_6_ vitamer concentration on PN concentration in the culture medium, cells were cultured in 6-well plates in complete DMEM (like #31966 (contains no PN) (Gibco), 10% FBS, 1% PS) containing 0, 10, 20, 40, and 60 μM PN (Sigma-Aldrich) for 96 h to confluency. In both experiments, medium was refreshed after 48 h and 72 h. After 96 h, culture medium was removed, cells were washed with 4 ml/well cold DPBS (BioWhittaker, Lonza), and cells were scraped in 0.6 ml/well of cold trichloroacetic acid (50 g/l) (Sigma-Aldrich) on ice. Cell extracts were vortexed vigorously and centrifuged at 16,200*g* for 5 min at 4 °C. Eighty microliters of the supernatant were analyzed as described in section ‘B6 vitamer analysis’.

For B_6_ vitamer analysis in protein-depleted and protein-enriched cellular fractions obtained by filtration, cells were cultured in 6-well plates in complete DMEM (#31966-021 (contains 19.4 μM PN, Gibco), 10% FBS, 1% PS) or complete DMEM with no vitamin B_6_ (like #31966 (contains no PN) (Gibco), 10% FBS, 1% PS) for 96 h to confluency. Culture medium was removed, cells were washed with 4 ml/well DPBS (BioWhittaker), and scraped in 0.5 ml/well of 40 mM Tris-phosphate buffer (pH 7.6, adjusted with 8.5% orthophosphoric acid) on ice. Cell suspensions from two wells were pooled yielding 3 × 1 ml per cell line and vitamin B_6_ concentration and were sonicated (Soniprep 150, MSE Centrifuges) for 30 s in the pulse mode (1 s on 1 s off, amplitude 10 μm) on ice. Next, 0.5 ml of cell lysate was loaded on Amicon Ultra 0.5 ml Centrifugal Filters (cutoff 10 kDa, Millipore, Merck) and centrifuged at 14,000*g* for 15 min at 4 °C. Eighty microliters of filtrate (protein-depleted) and 80 μl of concentrate (protein-enriched) were used for B_6_ vitamer analysis as described below.

### B_6_ vitamer analysis

Eighty microliters of sample (centrifuged trichloroacetic acid extracts of total cell or mitochondrial and cytosolic fractions or protein-depleted and protein-enriched cellular fractions) were mixed with 80 μl of trichloroacetic acid (50 g/l) containing isotopically labeled internal standards (47), incubated 15 min in the dark, and centrifuged at 16,200*g* for 5 min at 4 °C. B_6_ vitamers were analyzed with ultra-performance liquid chromatography tandem mass spectrometry (UPLC-MS/MS) as described (47). During all steps, samples were protected from light as much as possible. The content of B_6_ vitamers in total cell extracts was expressed in pmol/mg of cellular protein. The content of B_6_ vitamers in mitochondrial and cytosolic fractions was expressed in pmol/mg protein in the specific fraction. The content of B_6_ vitamers in protein-depleted/enriched fractions were corrected for differences in protein concentration in the initial total cell lysates (expressed for 1 mg/ml total cell protein). Protein and PLP recoveries in protein-depleted/enriched fractions were calculated according to the manufacturer’s protocol. Protein content was determined with the BCA protein assay kit according to the manufacturer’s protocol (Pierce, Thermo Fisher Scientific).

### Analysis of the dynamics of vitamin B_6_ metabolism

Cells were cultured in complete DMEM (#31966-021 (contains 19.4 μM PN, Gibco), 10% FBS, 1% PS) to >80% confluence. At time point 0 min, culture medium was replaced with complete DMEM (like #31966 (contains no PN) (Gibco), 10% FBS, 1% PS) containing 20 μM ^13^C_4_-pyridoxine HCl (Cambridge Isotope Laboratories), and cells were incubated for 0, 5, 10, 30, and 60 min. At the indicated time point, culture medium was removed, cells were washed with 4 ml/well DPBS (BioWhittaker), and scraped in 0.6 ml/well trichloroacetic acid (50 g/l) on ice. Cell extracts were vortexed and centrifuged at 16,200*g* for 5 min at 4 °C. Next, 80 μl of the supernatant was mixed with 80 μl of trichloroacetic acid (50 g/l), and B_6_ vitamers were analyzed according to UPLC-MS/MS method described (47) with modifications of MS parameters to measure ^13^C_4_-labeled B_6_ vitamers (Table S1). Fractional enrichment of ^13^C_4_-PN, ^13^C_4_-PNP, ^13^C_4_-PL, and ^13^C_4_-PLP was calculated by dividing the intensity of ^13^C_4_-labeled B_6_ vitamer by the sum of intensities of unlabeled plus ^13^C_4_-labeled B_6_ vitamer (total). Fractional enrichment time courses were fit to a mono-exponential function (Y = Y0 + (Plateau-Y0)∗(1-exp(-K∗x))) in GraphPad Prism version 8.3 for Windows, GraphPad Software, Boston, Massachusetts, http://www.graphpad.com), and the fractional turnover rate constant K was derived for each B_6_ vitamer and cell line.

### PNPO enzyme activity

Cells were cultured to confluency in 6-well plates in complete DMEM (#31966-021 (contains 19.4 μM PN, Gibco), 10% FBS, 1% PS), washed with 4 ml/well cold DPBS (BioWhittaker), and scraped in 1 ml/well of 40 mM Tris-phosphate buffer (pH 7.6, adjusted with 8.5% orthophosphoric acid). Cell suspensions were sonicated (Soniprep 150, MSE Centrifuges) for 30 s in the pulse mode (1 s on 1 s off, amplitude 10 μm) on ice. PNPO enzyme activity was assayed at 37 °C in an Eppendorf thermomixer at 500 rpm in five times–diluted cell lysates in assay mixture containing 40 mM Tris-phosphate buffer (pH 7.6), 0.4 μM or 2 μM PNP (Toronto Research Chemicals), and either no FMN (*i.e.*, only endogenous FMN present) or 1.5 μM FMN (Sigma-Aldrich) as described (23). Reaction was started by adding cell lysate to the assay mix. Samples were taken at 0, 15, and 30 min and quenched by either (1) adding equal volume (80 μl) of trichloroacetic acid (50 g/l) containing isotopically labeled internal standards (47) (assay with 0.4 μM PNP) or (2) four volumes of trichloroacetic acid (50 g/l) (64 μl to 16 μl sample) and subsequently 80 μl trichloroacetic acid (50 g/l) containing isotopically labeled internal standards (assay with 2 μM PNP). The dilution of the sample (5×) in the assay with 2 μM PNP was necessary to avoid interference of high PNP concentration in the UPLC-MS/MS analysis. Samples were vigorously vortexed, incubated for 30 min on ice in the dark, and centrifuged at 16,200*g* for 5 min at 4 °C. PLP in the supernatant (140 μl) was quantified using UPLC-MS/MS as described (47). PNPO activity was expressed in pmol PLP/min/mg total cell protein.

### GOT, CS, and PGI enzyme activity

All enzyme activities were measured in HEK293 cell total cell extracts and mitochondrial fractions in DPBS after sonicating for 30 s in the pulse mode (1 s on 1 s off, amplitude 10 μm) on ice. GOT (EC 2.6.1.1) activity was measured spectrophotometrically at 340 nm at 37 °C according to (48) using EnSight microplate reader (PerkinElmer). The assay mix contained 100 mM potassium phosphate buffer (pH 7.4), 0.2 mM NADH, 12 mM α-KG, 7.3 U/ml malate dehydrogenase (Roche), and sample. The reaction was started with 100 mM L-aspartate (pH 7.4) (Sigma-Aldrich). GOT activity was calculated using molar extinction coefficient of NADH (6.22 mM^−1^ cm^−1^). CS (EC 4.1.3.7) activity was determined spectrophotometrically at 412 nm at 37 °C in total cell lysates and mitochondrial fractions according to (49). The assay mix contained 100 mM Tris–HCl buffer (pH 8.1), 5 mM triethanolamine–HCl buffer (pH 8.0), 0.5 mM oxaloacetate, 0.1 mM dithionitrobenzoic acid, 0.1% (v/v) Triton X-100, and sample. The reaction was started with 0.3 mM acetyl-CoA (Sigma-Aldrich). CS activity was calculated using molar extinction coefficient of thionitrobenzoic acid (13.6 mM^−1^ cm^−1^). PGI (EC 5.3.1.9) was assayed at 340 nm at 37 °C as described (50). The assay mix contained 50 mM Tris–HCl buffer (pH 8.0), 5 mM MgCl_2_, 0.4 mM NADP^+^, 0.23 U/ml glucose-6-phosphate dehydrogenase (Roche), and sample. The reaction was started with 2 mM fructose-6-phosphate. PGI activity was calculated using molar extinction coefficient of NADPH (6.22 mM^−1^ cm^−1^). GOT, CS, and PGI activities were expressed in nmol/min/mg protein in specified cellular fraction.

### Western blotting

Cells were cultured in 6-well plates in complete DMEM (#31966-021, Gibco, containing 19.4 μM PN, 10% FBS 1% PS) to confluency, washed with 4 ml/well DPBS (BioWhittaker), and scraped in 0.3 ml of lysis buffer (50 mM Tris–HCl pH 8.0, 150 mM NaCl, 5 g/l sodium deoxycholate, 0.1% sodium dodecyl sulfate, 2 mM NaF, and protease inhibitor cocktail (1:200, Roche)) on ice. Cell lysates were solubilized for 2 h at 4 °C and centrifuged at 14,000*g* for 10 min at 4 °C. Mitochondrial and cytosolic fractions were obtained as described above. Equal amounts of protein in total cell lysates, mitochondrial, and cytosolic fractions were resolved on NuPAGE 4 to 12% Bis-Tris gels (Invitrogen, Thermo Fisher Scientific) and transferred to polyvinylidene difluoride membrane (Immobilon-P) with semidry blotting system (Novex, Invitrogen) following manufacturer’s recommendations. After blocking with tris-buffered saline (TBS) containing 0.1% Tween 20 (TBST) and 50 g/l skim milk (Nutricia) for 1 h at room temperature, the membranes were incubated overnight at 4 °C with primary rabbit polyclonal anti-PROSC (PLPHP) (1:1000, HPA023646, Sigma-Aldrich), rabbit polyclonal anti-CS (1:5000, ab96600, Abcam), or mouse monoclonal anti-GAPDH (1:5000, sc-365062, Santa Cruz Biotechnology) antibodies in TBST containing 10 g/l skim milk (Nutricia). Next, membranes were washed 3 × 5 min with TBST and incubated with a corresponding horseradish peroxidase–conjugated secondary antibody in TBST containing 5 g/l skim milk (Nutricia) for 1 h at room temperature. After the final wash of 3 × 5 min with TBST and 1 × 5 min with TBS, the immunocomplexes were detected using SuperSignal West Pico PLUS Chemiluminescent Substrate (cat. # 34580, Pierce, Thermo Fisher Scientific) and images were captured with the ChemiDoc MP imaging system (Bio-Rad Laboratories).

### PDXP inhibition and knockdown

For PDXP inhibition with high concentration of inorganic phosphate experiments, HEK293 cells were cultured in 6-well plates in complete DMEM (#31966-021 (contains 19.4 μM PN, Gibco), 10% FBS, 1% PS) to confluency. Culture medium was replaced with complete DMEM (like #31966 (contains no PN) (Gibco), 10% FBS, 1% PS) containing 20 μM ^13^C_4_-PN HCl and 80 mM NaH_2_PO_4_/Na_2_HPO_4_ (pH 7.4), and cells were incubated for 60 min. Culture medium was removed, and cells were washed with 4 ml/well cold DPBS (BioWhittaker) and scraped in 0.6 ml/well trichloroacetic acid (50 g/l) for B_6_ vitamer analysis.

For the *PDXP* knockdown experiments, HEK293 cells were seeded in 6-well plates (one plate per cell line and condition (not transfected, nontargeting negative control siRNA and *PDXP* siRNA)) and cultured to ∼50% confluence in complete DMEM (#31966-021 (contains 19.4 μM PN, Gibco), 10% FBS, 1% PS). Cells were transfected in OptiMEM medium (#31985-047, Gibco) with either nontargeting negative control siRNA (final concentration 15 nM) (ON-TARGETplus nontargeting control pool, cat. #D-001810-10-05, Horizon Discovery) or human *PDXP* siRNA (final concentration 15 nM, ON-TARGETplus SMART pool, #L-017120-00-0005, Horizon Discovery) using Lipofectamine RNAiMAX reagent (Invitrogen, Thermo Fisher Scientific) according to the manufacturer’s protocol. Nontransfected cells were also refreshed with OptiMEM medium. After 24 h, all cells were refreshed with complete DMEM (#31966-021 (contains 19.4 μM PN, Gibco), 10% FBS, 1% PS) and incubated for another 24 h (total duration of *PDXP* knockdown 48 h). Culture medium was removed, and cells were washed with 4 ml/well cold DPBS (BioWhittaker) and harvested for total RNA isolation and real-time PCR and B_6_ vitamer analysis.

### RNA isolation and real-time PCR

Total RNA was isolated with Tri reagent (Sigma-Aldrich) according to the manufacturer’s protocol. One microgram of total RNA was reverse transcribed to complementary DNA using M-MLV reverse transcriptase (Invitrogen) according to the manufacturer’s protocol. RT PCR was performed with a StepOne Real-Time PCR System (Applied Biosystems) using SYBR Select Master Mix (Applied Biosystems) and primers listed in Table S2. The mRNA levels of *PDXP* were normalized to the mRNA level of ribosomal protein lateral stalk subunit P0 (*RPLP0*) and expressed relative to the corresponding control (calculated according to the ΔΔCt method).

### Amino and organic acid analysis

Cells were cultured in 6-well plates in complete DMEM (#31966-021 (contains 19.4 μM PN, Gibco), 10% FBS, 1% PS) 96 h to confluency. Culture medium was removed, and cells were washed with 4 ml/well cold DPBS (BioWhittaker) and scraped in 0.5 ml/well of cold 100% methanol (kept on dry ice). Cell extracts were vortexed vigorously and centrifuged at 16,200*g* for 5 min at 4 °C. Amino acid analysis was performed in 40 μl of undiluted supernatants using UPLC-MS/MS method as described (51). Amino acid concentrations were expressed in nmol/mg cellular protein. Organic acids were measured in methanol extracts prepared for amino acid analysis using UPLC-MS/MS method as described (52).

### Statistical analysis

All data are presented as means ± SD. Data from the two HEK293 control clonal cell lines were pooled, since no significant differences in any of the measured parameters were observed. Statistical analysis was performed using GraphPad Prism version 8.3 for Windows, GraphPad Software, Boston, Massachusetts, http://www.graphpad.com). The data were analyzed using either one-way ANOVA followed by Tuckey’s post hoc test or unpaired Student’s *t* test. The use of the specific test is indicated in the figure legends. The level of significance was set at *p* < 0.05.

## Data availability

The authors confirm that the data supporting the findings of this study are available within the article and its supporting information.

## Supporting information

This article contains supporting information.

## Conflict of interest

The authors declare that they have no conflicts of interest with the contents of this article.
