# Supplementary material for: Maintenance of cellular vitamin B6 levels and mitochondrial oxidative function depend on pyridoxal 5′-phosphate homeostasis protein
Source: J Biol Chem. 2023 Jul 13;299(9):105047. doi: 10.1016/j.jbc.2023.105047 (PMC10463200; doi:10.1016/j.jbc.2023.105047)
Supplement: Supporting information [file mmc1.docx]

**Supporting information**

**Maintenance of cellular vitamin B_6_ levels and mitochondrial oxidative function depend on pyridoxal 5’-phosphate homeostasis protein (PLPHP)**

Jolita Ciapaite, Carlo W. T. van Roermund, Marjolein Bosma, Johan Gerrits, Sander M. Houten, Lodewijk IJlst, Hans R. Waterham, Clara D. M. van Karnebeek, Ronald J. A. Wanders, Fried J.T. Zwartkruis, Judith J. Jans, Nanda M. Verhoeven-Duif

**Table of contents**

Table S1………………………………………………………………………………………………………2

Table S2………………………………………………………………………………………………………3

Figure S1……………………………………………………………………………………………………..4

Figure S2……………………………………………………………………………………………………..5

Figure S3……………………………………………………………………………………………………..6

Figure S4………………………………………………………………………………………………….….7

Figure S5………………………………………………………………………………………………….….9

Figure S6…………………………………………………………………………………………………….10

**Table S1 Mass spectrometry parameters for quantification of B_6_ vitamers.**

| **Compound name** | **Parent ion (m/z)** | **Daughter ion (m/z)** | **Cone voltage (V)** | **Collision energy (V)** |
| --- | --- | --- | --- | --- |
| Pyridoxal | 168 | 150 | 16 | 12 |
| Pyridoxamine | 169 | 134 | 16 | 22 |
| Pyridoxine | 170 | 134 | 18 | 20 |
| d_3_-pyridoxal | 171 | 153 | 15 | 12 |
| ^13^C_4_-pyridoxal | 172 | 154 | 16 | 12 |
| ^13^C_4_-pyridoxamine | 173 | 138 | 16 | 22 |
| ^13^C_4_-pyridoxine | 174 | 138 | 18 | 20 |
| Pyridoxic acid | 184 | 148 | 18 | 18 |
| d_2_-pyridoxic acid | 186 | 150 | 18 | 18 |
| ^13^C_4_-pyridoxic acid | 188 | 152 | 18 | 18 |
| Pyridoxal 5'-phosphate | 248 | 150 | 28 | 14 |
| Pyridoxamine 5'-phosphate | 249 | 232 | 24 | 14 |
| Pyridoxine 5'-phosphate | 250 | 134 | 22 | 20 |
| d_3_-pyridoxal 5'-phosphate | 251 | 153 | 22 | 14 |
| ^13^C_4_-pyridoxal 5'-phosphate | 252 | 154 | 28 | 14 |
| ^13^C_4_-pyridoxamine 5'-phosphate | 253 | 236 | 24 | 14 |
| ^13^C_4_-pyridoxine 5'-phosphate | 254 | 138 | 22 | 20 |

**Table S2 List of primers used in RT PCR.** Primers were designed using Primer Express software version 3.0 (Applied Biosystems) using indicated NCBI mRNA reference sequences.

| **Oligonucleotide name** | **Accession number** | **Sequence** |
| --- | --- | --- |
| PDXP FW | NM_020315.4 | ACCTCGTGCCCCATTACTATGT |
| PDXP RV | NM_020315.4 | TCAGTCCTCCAACCCCTCTGT |
| RPLP0 FW | NM_053275.3 | CCATTGAAATCCTGAGTGATGTG |
| RPLP0 RW | NM_053275.3 | TCGCTGGCTCCCACTTTG |

**Figure S1**

**Figure S1 No co-immunoprecipitation of PLPHP with PNPO in HEK293 cells.** Wild type (WT), PNPO KO1 and PLPHP KO1 HEK293 cells we lysed in buffer containing 40 mM HEPES (pH 7.5), 120 mM NaCl, 1 mM EDTA, 0.3% CHAPS and protease inhibitors. PNPO was immunoprecipitated (IP) with polyclonal goat anti-PNPO IgG (sc-82319, Santa Cruz Biotechnology) using SureBeads protein G magnetic beads (Bio Rad). In PNPO antibody (Ab) control, antibody was omitted (WT cell lysate + SureBeads protein G magnetic beads). Mock IP was done with polyclonal goat anti-HSP 90β IgG (sc-1057, Santa Cruz Biotechnology). Samples of PNPO IP’s, mock IP, control IP and total cell lysates prior to IP were resolved with SDS PAGE, transferred to PVDA membranes and probed with goat anti-PNPO IgG (sc-82319, Santa Cruz Biotechnology), rabbit anti-PROSC (PLPHP) antibody (1:1000, HPA023646, Sigma-Aldrich) and rabbit anti-actin (1:20000, A5060, Sigma-Aldrich).

**Figure S2**

A

B

**Figure S2 Riboflavin supplementation for 96 hours has no effect on B_6_ vitamer profiles in control and PLPHP deficient fibroblasts (A) and HEK293 cells (B).** Cells were seeded on 6-well plates and cultured for 96 h in either complete DMEM ( contains 1.06 µM riboflavin) or complete DMEM supplemented with 4 µM riboflavin (total 5.06 µM riboflavin). Culture medium was refreshed after 48 and 72 h. Data are means from n=3-6 biological replicates per cell line and condition, ±SD.

**Figure S3**

**Figure S3 4-Pyridoxic acid concentration in fibroblasts and HEK293 cells, and yeast.**

A) Intracellular concentration of 4-pyridoxic acid (4-PA) in control and PLPHP deficient HEK293 cells cultured in complete DMEM containing no vitamin B_6_, 20 μM PN, 20 μM PL or 20 μM PM for 96 hours. Data are means from n=5-10 biological replicates per cell line and condition, ±SD.

B) Intracellular concentration of 4-PA in control and PLPHP deficient patient fibroblasts cultured in complete DMEM containing no vitamin B_6_, 20 μM PN, 20 μM PL or 20 μM PM for 96 hours. Data are means from n=6-9 biological replicates per cell line and condition, ±SD.

C) Intracellular 4-PA concentrations in wild type (WT) and YBL036C deficient (*ybl036c∆*) yeast grown on glucose, oleate or ethanol as carbon source. Data are means from n=2 independent experiments, ±SD.

D) Initial B_6_ vitamer concentrations in yeast culture media containing glucose, oleate and ethanol. Data are means from n=2 independent experiments, ±SD.

**Figure S4**

**Figure S4 The effect of PLPHP deficiency on the dynamics of vitamin B_6_ metabolism.**

**A)** The time course of ^13^C_4_ labeling of PN, PNP, PLP and PL in response to 20 μM ^13^C_4_-labeled pyridoxine (^13^C_4_-PN) supplementation in control and PLPHP deficient fibroblasts. Cells were plated in 6-well plates and cultured in complete DMEM (Gibco, cat. # 31966-021, contains 19.4 μM PN, 25 mM glucose, 1 mM pyruvate, 4 mM L-alanyl-L-glutamine; plus 10% FBS, 1% PS) to near confluency. Culture medium was replaced with complete DMEM of identical composition, except that unlabeled pyridoxine was replaced with 20 μM ^13^C_4_-PN and cells were incubated for 0, 5, 10, 30 and 60 min. At indicated time points cells were washed with 4 ml/well DPBS, 0.6 ml/well of trichloracetic acid (TCA, 50 g/l) was added and cells were scraped on ice. Centrifuged TCA extracts were used for B_6_ vitamer analysis. Data are means from n=3 per cell line, ±SD.

**B)** Fractional enrichment of ^13^C_4_-labeled PN, PNP, PLP and PL in response to 20 μM ^13^C_4_-labeled pyridoxine (^13^C_4_-PN) supplementation in control and PLPHP deficient fibroblasts. Fractional enrichment was calculated by dividing intensity of ^13^C_4_-labeled B_6_ vitamer by the sum of intensities of total B_6_ vitamer (unlabeled plus ^13^C_4_-labeled). The data were fit to a mono-exponential function in GraphPad Prism 8.3 (Y=Y0 + (Plateau-Y0)*(1-exp(-K*x)), shown as lines) from which fractional turnover rate constant K was derived for each B_6_ vitamer and cell line. Data are means from n=3 per cell line, ±SD.

**C)** The time course of ^13^C_4_ labeling of PN, PNP, PLP and PL in response to 20 μM ^13^C_4_-labeled pyridoxine (^13^C_4_-PN) supplementation in control and PLPHP deficient HEK293 cells. Experimental setup was the same as described in panel A. Data are means from n=6-12 per cell line, ±SD.

**D)** Fractional enrichment of ^13^C_4_-labeled PN, PNP, PLP and PL in response to 20 μM ^13^C_4_-labeled pyridoxine (^13^C_4_-PN) supplementation in control and PLPHP deficient HEK293 cells. Calculations of fractional enrichments, data fitting and calculations of fractional turnover rate constant K were the same as in panel B. Data are means from n=6-12 per cell line, ±SD.

**Figure S5**

**Figure S5 Increased pyridoxine (PN) consumption and pyridoxal (PL) production in PLPHP deficient HEK293 cells.** Cells were plated in 6-well plates and cultured in complete DMEM (Gibco, cat. # 31966-021, contains 19.4 μM PN, 25 mM glucose, 1 mM pyruvate, 4 mM L-alanyl-L-glutamine; plus 10% FBS, 1% PS). Medium samples were taken 24 and 48 h after refreshing the culture medium. Data are means from n=3-6 per cell line and time point, ±SD. ***p<0.001 compared to control cells at the same time point (unpaired t-test).

**Figure S6**

**Figure S6 Amino acid profiles in control and PLPHP deficient HEK293 cells**. Cells were plated in 6-well plates and cultured in in complete DMEM containing ~19.4 µM pyridoxine. (cat. # 31966, Gibco), 10% fetal bovine serum and 1% penicillin and streptomycin for 96 hours. Cells were harvested 1 h after refreshing culture medium. Data are means from n=6-12 per cell line, ±SD. *p<0.05, **p<0.01, ***p<0.001 and ****p<0.0001 compared to control cells.
